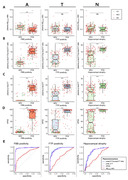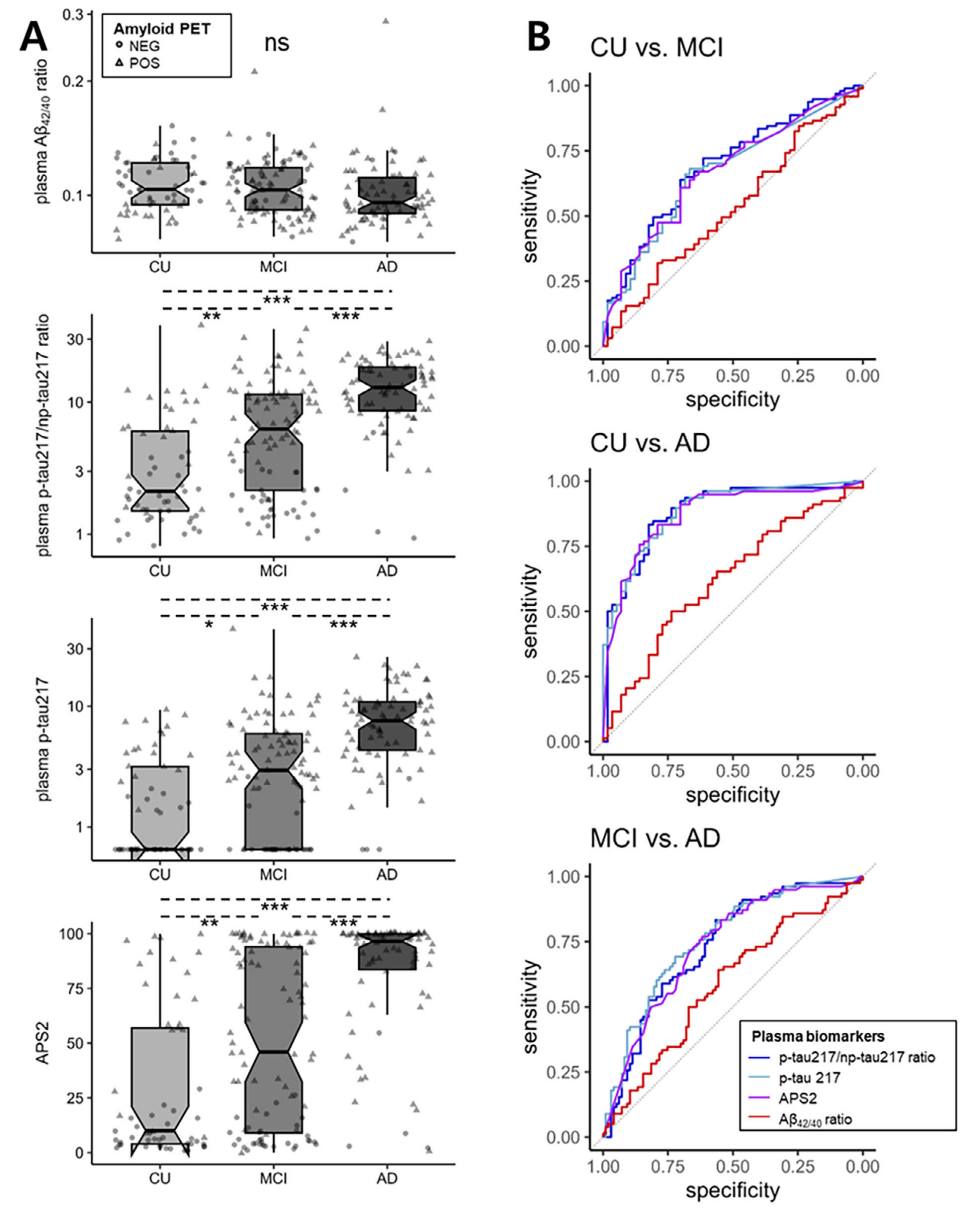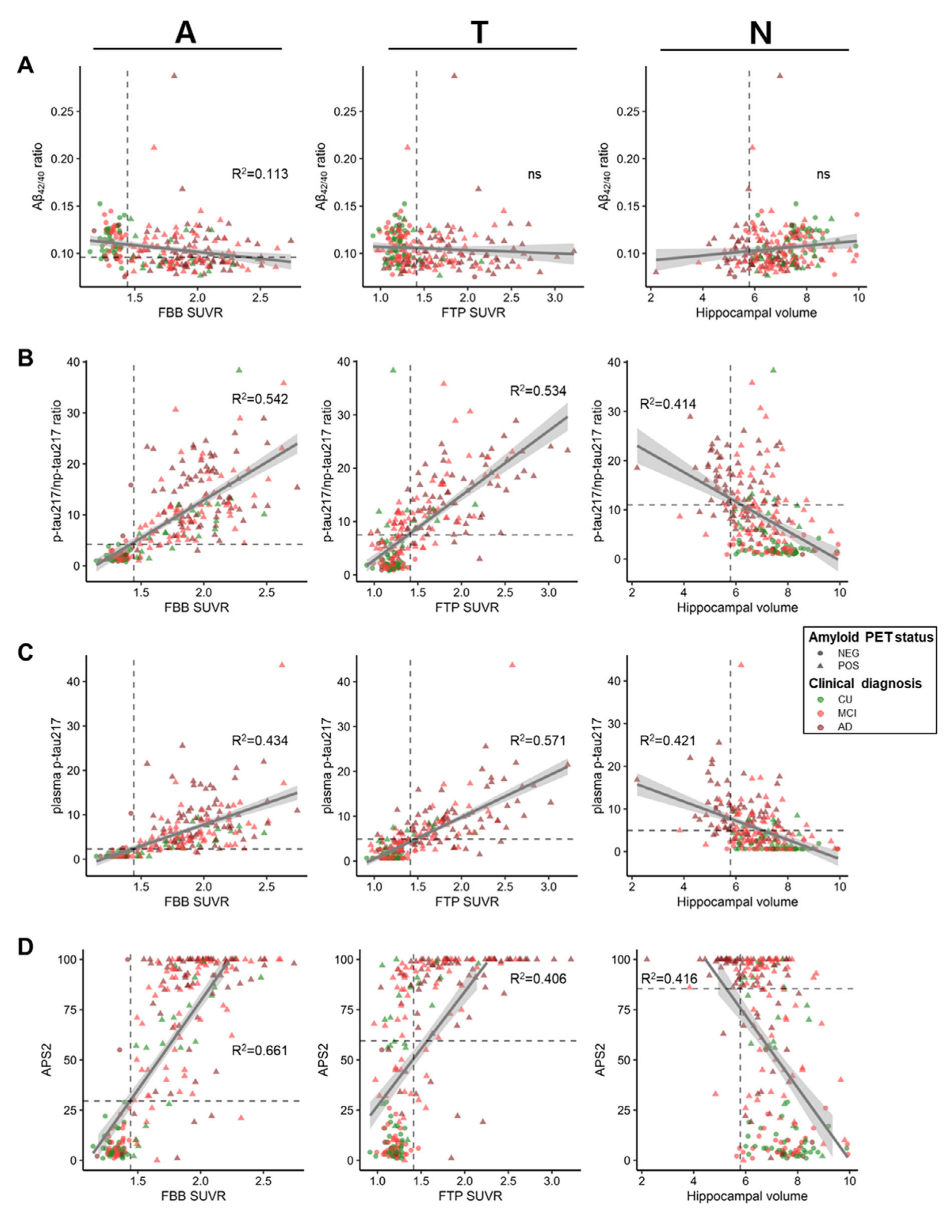# Diagnostic Performance of Plasma Biomarkers for A/T/N Image Classification in Alzheimer's disease

**DOI:** 10.1002/alz.088471

**Published:** 2025-01-09

**Authors:** Han‐Kyeol Kim, Jae Hoon Lee, Joong‐Hyun Chun, Jeong‐Ha Lee, Tim West, Kristopher M. Kirmess, Philip B. Verghese, Joel B. Braunstein, Daniel Connell, Young Hoon Ryu, Chul Hyoung Lyoo, Hanna Cho

**Affiliations:** ^1^ Wonju Severance Christian Hospital, Yonsei University Wonju College of Medicine, Wonju Korea, Republic of (South); ^2^ Gangnam Severance Hospital, Yonsei University College of Medicine, Seoul Korea, Republic of (South); ^3^ Severance Hospital, Yonsei University College of Medicine, Seoul Korea, Republic of (South); ^4^ C2N Diagnostics, LLC, Saint Louis, MO USA

## Abstract

**Background:**

We aimed to validate the ability of Alzheimer’s disease plasma biomarkers to predict clinical diagnosis and the ability to predict image‐based A/T/N biomarkers positivity in Asian population. We also sought to define the optimal cut point for each biomarker.

**Method:**

232 participants were enrolled, all of whom underwent ^18^F‐florbetaben (FBB), ^18^F‐flortaucipir (FTP) PET, volumetric MRI, and neuropsychological assessments. Their plasma samples, collected at the time of the imaging scans and stored at ‐80 degrees Celsius, were tested for plasma p‐tau217/np‐tau217 ratio, p‐tau217, Aβ_42/40_ ratio, and APS2 score at C2N Diagnostics. FBB and FTP positivity were determined based on the SUVR value through a Gaussian mixture model. The presence of hippocampal atrophy was defined as the mean minus 2 standard deviations of amyloid‐negative cognitive unimpaired participants. ROC analysis was conducted to assess the predictive ability and determine the optimal cut‐off point.

**Result:**

The ability to predict FBB PET positivity was excellent for p‐tau217/np‐tau217 ratio, p‐tau217, and APS2 (AUC=0.964, 0.963, and 0.958, respectively) while Aβ_42/40_ ratio showed lower performance with an AUC of 0.726. The Aβ_42/40_ ratio did not have the ability to predict FTP positivity, but p‐tau217/np‐tau217 ratio, p‐tau217, and APS2 all exhibited excellent performance with AUC = 0.926, 0.937, and 0.907, respectively. The Aβ_42/40_ ratio also did not have the ability to predict hippocampal atrophy, while p‐tau217/np‐tau217 ratio, p‐tau217, and APS2 demonstrated good performance (AUC=0.809, 0.815, and 0.811, respectively). Similarly, only the p‐tau217/np‐tau217 ratio, p‐tau217, and APS2 had the ability to differentiate clinical diagnosis while Aβ_42/40_ ratio failed to distinguish. The AUC‐ROC for p‐tau217/np‐tau217, p‐tau217, and APS2 all showed no significant difference in DeLong’s test when predicting X Y Z. The optimal cutoffs for predicting amyloid, tau and neurodegeneration by p‐tau217/np‐tau217 ratio, p‐tau217 and APS2 showed increases following the progression of AD.

**Conclusion:**

The plasma biomarker using p‐tau217 reflects the clinical status well on its own and is excellent at predicting the positivity of image‐based biomarkers and diagnostic performance. Among the image‐based A/T/N biomarkers, the predictive power was excellent in the order of A, T, and N. The p‐tau217 alone was not inferior to the p‐tau217/np‐tau217 ratio or APS2